# The Association between Longest-Held Lifetime Occupation and Late-Life Cognitive Impairment: Korean Longitudinal Study of Aging (2006–2016)

**DOI:** 10.3390/ijerph17176270

**Published:** 2020-08-28

**Authors:** Hye-Jin Kim, Jin-Young Min, Kyoung-Bok Min

**Affiliations:** 1 Department of Preventive Medicine, College of Medicine, Seoul National University, 103 Daehak-ro, Jongno-gu, Seoul 08826, Korea; okimhj@snu.ac.kr; 2 Institute of Health and Environment, Seoul National University, Seoul 08826, Korea; yaemin00@snu.ac.kr

**Keywords:** longest-held lifetime occupation, cognitive impairment, cognitive function, gender differences

## Abstract

The association between longest-held lifetime occupation and late-life cognitive impairment: Korean Longitudinal Study of Aging (2006–2016). *Backgrounds*: Our study hypothesized that occupation in adulthood may be one of the modifiable factors in cognitive performance. This follow-up study aimed to examine whether there was an association between the longest-held occupation in a lifetime and cognitive impairment. *Methods*: This study used data from the 2006, 2012, and 2016 waves of the Korean Longitudinal Study of Aging, and a total of 1733 subjects aged over 65 were included. Longest-held occupation in a lifetime was classified into blue-collar, pink-collar, and white-collar. Cognitive function was evaluated using the Korean version of the Mini-Mental State Examination. *Results*: In males, no significant associations were observed. In females, on the contrary, risk of cognitive impairment in the blue-collar occupation was consistently higher than in the white-collar occupation over the 10-year follow-up (2006, OR = 2.49, 95% CI 1.05–5.88; 2016, OR = 2.17, 95% CI 1.02−4.65). *Conclusions*: Lifetime occupation should be taken into consideration in the process of screening for cognitive decline in the elderly, especially females. This study needs to be interpreted cautiously in view of inherent data and methodological limitations.

## 1. Introduction

With the rapid population aging, cognitive health is of critical importance for maintaining independence and quality of life in old age [1]. Cognitive function tends to decline after peaking at approximately age 40 and leads to more significant declines after the age of 80 [2,3,4,5]. It is known that 30–40% of cases with mild cognitive impairment consequently progress to more severe conditions such as dementia [6]. As cognitive impairment and dementia undermine overall daily functioning and cause disability, in older adults, even delaying the onset of cognitive decline is vital [7]. There is a consensus that a substantial proportion of cases of cognitive impairment is preventable [8]. The early onset of cognitive deficits could be attributable to the following potentially modifiable factors: Low educational attainment, housing environment, smoking, dietary intake (e.g., Mediterranean diet, vegetable, and ω-3 fatty acid intake), cognitive inactivity, and clinical condition (e.g., diabetes, midlife hypertension and obesity, and depression) [9,10]. However, few studies demonstrated the effect of occupation on cognitive decline. Therefore, it is necessary to identify job-related modifiable risk factors for cognitive impairment.

Occupation is highly dependent on educational attainment in early life, determines income and economic resources, and affects healthy lifestyles [11,12,13]. Occupation is considered as an important predictor of cognitive decline in later life [12,14], because of the disparities in working environment and variations in cognitive demands across occupations [15]. Employees in manual (or blue-collar) occupations than non-manual occupations are more likely to experience a variety of deleterious work factors such as greater job-strain, lack of social support, occupational mobility, job insecurity, physical/chemical/ergonomic hazards at work, and low wage income, which may cause occupational health disparities [16,17,18]. Several studies have shown that manual workers are vulnerable to poorer physical (e.g., cardiovascular disease, musculoskeletal pain, sickness absence) and mental health condition (e.g., stress, depression, suicide ideation) than non-manual workers [19,20]. There is evidence indicating the role of occupation in subsequent cognitive deterioration. Individuals holding an inferior position in their job have higher cognitive impairment risks [21], and occupation-related cognitive requirement and occupational complexity are associated with late-life cognitive decline [22]. Prior research also demonstrated the relation between mental work demands and cognitive change over age [23]. As most people spend a substantial portion of their lives at work [24]. The workplace environment has a greater effect on workers’ cognitive health [25]. Manual workers are frequently exposed to industrial toxicants and neurotoxic materials in the work environment [8,26]. While those in white-collar occupations tend to constantly exert themselves intellectually [27,28]. Moreover, the longest-held lifetime occupation is a significant discriminator of socioeconomic differentials and reflects the individual’s accumulated lifetime employment, working conditions, and other factors related to occupation [21]. Lifelong working in their principal job, individuals would be consistently exposed to intellectually stimulating activities, psychosocial workplace conditions, income-related healthy lifestyles, and environmental neurotoxicants in their workplace, which may trigger cognitive decline in later life [26]. For older people, health status is not the result of events occurring over a short period but the accumulation of health behaviors throughout the life course. Thus, the cumulative effect of job experience would produce health disparities in old age [29,30]. 

From employment to retirement, males and females experience various social positions and social roles throughout their lives [31]. There is a gender differential in labor compensation, occupied industrial sectors, wages, and household roles [32]. Females have concentrated in female-dominated occupations, and these jobs pay less than male-dominated occupations [33]. Moreover, female workers are highly employed in jobs requiring nurturant social skills such as nursing, teaching, child-care work, and counseling. They have limited internal mobility in their job and low capital intensity compared to male workers [34]. Thus, there may be a different association between lifetime occupation on later health status between genders. 

Based on this background, this study aimed to investigate whether there is an association between the longest-held lifetime occupation and cognitive impairment in the Korean elderly. Specifically, considering gender differences in the occupational experience, we estimated the risk of cognitive impairment across the type of longest-held lifetime occupation for both genders, respectively.

## 2. Methods

### 2.1. Data Source and Study Population

**Figure 1:**
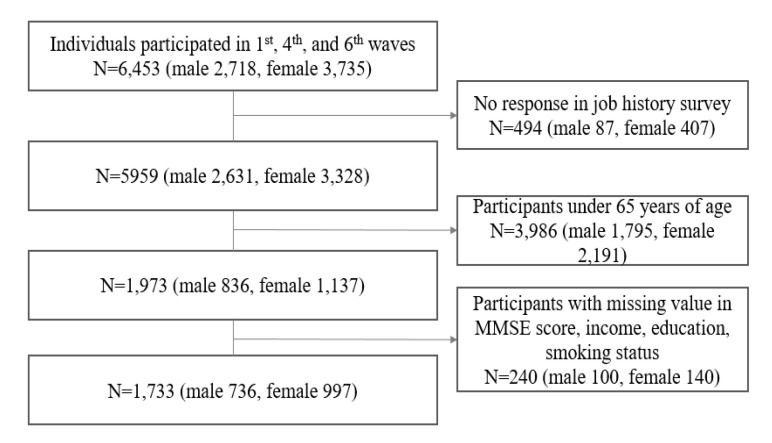
A flow chart of study population (*N* = 1733; male 736, female 997).

This study used the secondary data source of the 1st, 4th, and 6th waves of the Korean Longitudinal Study of Aging (KLoSA; 2006, 2012, and 2016). We included information regarding demographic factors, socioeconomic status, health behaviors, occupation, cognitive function, and the job history dataset. The KLoSA, a nationally representative longitudinal study, is a biennial undertaking of the Korean Labor Institute of the Ministry of Labor. It uses multistage, stratified cluster sampling to obtain representative estimates for non-institutionalized residents in Korea [35]. The initial survey in 2006 covered 10,245 adults aged 45 years and older. In the following ten years (2006–2016), the follow-up rate was 78% (*N* = 7015). Only individuals who were enrolled in all the follow-up cohorts were included in the current study (*N* = 6453; male 2718, female 3735), and we excluded the participants with no response in the job history survey (*N* = 494; male 87, female 407). Since our research focused on the elderly, 3986 subjects aged 64 or below were excluded (*N* = 3986; male 1795, female 2191). In addition, 240 subjects with missing values in MMSE scores, income, education, and smoking status were excluded (*N* = 240; male 100, female 140). Therefore, the final sample comprised 1733 subjects: 736 males and 997 females (Figure 1). 

### 2.2. Longest-Held Job in a Lifetime

The occupational history dataset included profession-related details over the lifetime, right from the participants’ first job in life: Calendar period at hire, calendar period at the exit for each job, and calendar time for unemployment. Occupation generally presents workplace exposure [4,36,37,38] and the longest-held occupation reflects the worker’s accumulated lifetime working conditions and work-related risk exposures [21]. Longest-held occupation in a lifetime was determined by the occupation in which an individual was employed for the longest duration in adulthood. Individuals who had never been employed were excluded. Longest-held occupation in a lifetime was categorized into three types: White-collar, blue-collar, and pink-collar. White-collar refers to clerical and kindred work and included assistant to executives, officials, business, and professional workers [39]. Pink-collar was generally defined as retail and service industry work [38], and blue-collar refers to the industry with manual labor [40]. We also categorized managers, professionals, and clerks as white-collar workers; service and sales workers as pink-collar workers; and skilled agricultural, forestry and fishery workers, craft and trades workers, equipment and machine operation and assembly workers, elementary workers, and armed forces as blue-collar workers [41].

### 2.3. Cognitive Impairment

Cognitive function was determined by the Korean version of the Mini-Mental State Examination (K-MMSE). The K-MMSE consists of 19 items, including orientation to time and space (five items), memory registration and recall (two items), concentration and calculation (five items), language (six items), and visuospatial construction (one item). The scores range from 0 to 30, with lower scores indicating lower cognitive function. Individuals with less than 24 points for K-MMSE scores were classified into cognitive impairment, and equal or more than 24 points were classified into a high cognitive function [20,42]. The cognitive impairment (MMSE) was assessed approximately 4.7 years after the retirement in male (blue-collar, 3.9 years; pink-collar, 3.5 years; and white-collar, 7.3 years), and approximately 12.9 years in female (blue-collar, 12.5 years; pink-collar, 12.5 years; and white-collar 20.9 years).

### 2.4. Other Variables of Interests

Covariates included sociodemographic occupational factors, and health-related factors, which can potentially confound the relationship between longest-held occupation and cognitive impairment in later life. Sociodemographic factors included age (65–74 years or 75 years and older), marital status (married, divorced/widowed, or never married), living alone (yes or no), educational level (elementary or lower, middle/high school, or college or higher), yearly income ((10,000,000 KRW (approximately <9000 USD), 10,000,000−25,000,000 KRW (approximately 9000–22,500 USD), or >25,000,000 KRW (approximately 22,500 USD)). Occupational factors included work duration (mean = 31.65, SD = 21.60 in female; mean = 40.30, SD = 15.35 in female) (gender—specific median values; high (male > 39 years and female > 29 years) or low (male ≤ 39 years and female ≤29 years)), and time spent from retirement to examination of cognitive function (gender-specific median values; high (male >1 year and female >8 years) or low (male ≤1 year and female ≤8 years)). Health-related factors included smoking status (never, former, or current), alcohol consumption (never, former, or current), muscle strength (gender-specific median values; high (male > 28.75 kg and female > 17.0 kg) or low (male ≤ 28.75 kg and female ≤17.0 kg)), physical activity (yes or no), well-being (yes (general life satisfaction score ≥50) or no (general life satisfaction score <50)), and chronic diseases (yes or no) such as history of heart disease, diabetes, psychiatric diseases, and stroke.

### 2.5. Statistical Analysis

All the analyses were gender stratified because of the potential gender disparities in the association between occupation and cognitive deficits. Differences in subjects’ general characteristics by the longest-held lifetime occupation were tested using ANOVA and chi-square tests. Moreover, if there are more than 20% of the expected cell frequencies <5, we conducted Fisher’s exact test (differences in marital status by longest-held occupation in male; differences in marital status, education, smoking status, and alcohol consumption). Trajectories of K-MMSE scores from 2006 to 2016 by the longest-held lifetime occupation were presented and the Cochran-Armitage trend test was used to determine whether the K-MMSE scores demonstrated any wave-related trends. Furthermore, differences in the proportions of subjects with cognitive impairment across the longest-held lifetime occupation in each wave were examined using chi-square tests. Unadjusted and adjusted logistic regression models were used to examine the association between the longest-held lifetime occupation and cognitive impairment in all three waves (2006, 2012, and 2016). The regression models were adjusted for age, marital status, education, income, cigarette smoking, alcohol drinking, physical activity, muscle strength, the presence of chronic diseases, work duration, time spent after retirement, well-being, and living alone. All analyses were conducted using the SAS 9.4 software (SAS Institute, Cary, NC, USA), and *p* ≤ 0.05 was considered to indicate statistical significance.

### 2.6. Ethical Approval 

The study protocol of this cohort study was reviewed and approved by the Institutional Review Board of Seoul National University/Seoul National University Hospital (IRB No. E-1905-076-1033).

## 3. Results

Table 1 shows the characteristics of the study population according to the longest-held lifetime occupation by gender. In males, a higher proportion of blue and pink-collar workers than white-collar workers were divorced/widowed, had a low educational level, were current smokers, and did not engage in daily physical activity. Blue and pink-collar workers were more likely to have low muscle strength, to live no well-being life, and to spend a longer time after retirement. In females, a higher proportion of blue and pink-collar workers than white-collar workers were older and current drinkers, blue and pink-collar workers were more likely to have a low educational level, no physical activity, and low muscle strength and to spend a longer time after retirement. For both males and females blue-collar workers were more likely to work longer than white and pink-collar workers.

**Figure 2:**
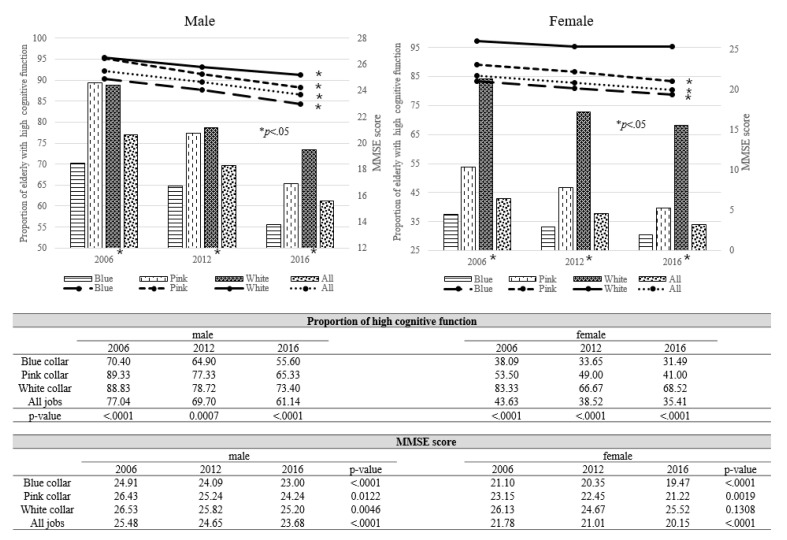
Gender-specific proportion of high cognitive function (K-MMSE ≥ 24) and mean scores of K-MMSE according to the occupational types over 10 years (2006, 2012, and 2016). (In male, the prevalence of high cognitive function and K-MMSE scores in all occupations decreased over the following ten years with the lowest cognitive function in blue-collar workers; in female, the proportion of subjects with high cognitive function decreased over the years with the lowest percentage of high cognitive function in blue-collar workers. K-MMSE scores significantly decreased over the years in blue and pink-collar workers.).

Figure 2 depicts the gender-specific proportion of high cognitive function (K-MMSE ≥ 24) and mean K-MMSE scores according to the occupational types over ten years. In males, the prevalence of high cognitive function in all occupations was 77.04% in 2006, decreasing over the following ten years. The percentage of high cognitive function was the lowest in blue-collar workers (55.6–70.4%). Moreover, K-MMSE scores decreased significantly over the years in all occupational types (from 25.48 to 23.68), (*p* < 0.0001). The gap in K-MMSE scores between blue-collar and white-collar workers increased over 10 years (from 1.62 to 2.2). Similarly, in females, the proportion of subjects with high cognitive function decreased over the years (from 43.63% to 35.41%). The lowest percentage of high cognitive function was observed in blue-collar workers (31.49–38.09%). Further, K-MMSE scores significantly decreased over the years in blue (from 21.1 to 19.47) (*p* < 0.0001) and pink-collar workers (from 23.15 to 21.22) (*p* = 0.0019). The gap in K-MMSE scores between blue and white-collar workers increased slightly over the years (from 5.03 to 6.05).

Table 2 shows the prevalence of cognitive impairment and unadjusted and adjusted odds ratios (ORs) of cognitive impairment by the longest-held lifetime occupation in males over ten years (2006, 2012, and 2016). In 2006, the proportion of subjects with cognitive impairment was highest in blue-collar workers (29.60%) and lowest in pink-collar workers; this proportion increased over the years. 

For females in Table 3, the proportion of subjects with cognitive impairment was the highest in blue-collar workers and the lowest in white-collar workers in 2006. The prevalence of cognitive impairment increased gradually over the years for each occupation type. In the full adjusted model, the association between the longest-held occupation and cognitive impairment was significant in 2006 (OR = 2.49, 95% CI 1.05–5.88) and maintained after 10 years (2016) (OR = 2.17, 95% CI 1.02–4.65).

## 4. Discussion

Our study demonstrated the 10-year association between the longest-held lifetime occupation on cognitive impairment in elderly Koreans. In males, no significant risks of cognitive impairment were observed in all three waves. In females, there was a 2.49-fold increased risk of cognitive impairment in blue-collar workers compared to white-collar workers. This risk was maintained in the following wave, at 2.17-fold higher in 2016. As per these results, in individuals aged 65 and older, working in blue-collar during lifetime was associated with decreased cognitive function, and the longitudinal effect was significant only in females.

To our knowledge, few studies have conducted follow-ups to determine whether the longest-held lifetime occupation consistently affects cognitive functioning in later life. However, several scholars have attempted to clarify the relationship between longest-held occupation in a lifetime and cognitive decline. Although it is difficult to summarize previous results because of classification-related variations, they commonly concluded that, in comparison with former white-collar workers, blue-collar workers are vulnerable to cognitive declines in old age [12,21,43]. For instance, Chung-Yi Li et al. (2002), Cat Tuong Nguyen et al. (2008), and Jean-Francois Dartigues et al. (1992) reported that manual workers (e.g., plant and machine operators, assemblers, and elementary workers), workers in the agriculture, forestry, and fishery sectors, and housewives had an elevated risk of cognitive impairment as compared to non-manual workers (e.g., business executives, managers or government administrators, and workers employed in intellectual occupations) [12,21,43]. Additional studies have demonstrated that the risk of dementia and Alzheimer’s disease, the most severe expressions of cognitive impairment, were greater among manual workers involved in goods production, farmers, and domestic service employees than among professionals and managers [14,26]. 

Our results support and expand these prior findings. Several hypotheses would be useful in understanding the association between the longest-held lifetime occupation and risk of cognitive impairment. First, occupations that involve frequent and intense intellectual challenges and cognitive requirements produce greater intellectual stimulation in the workplace [24,28]. Further, these occupational activities can increase neural growth and synaptic density and compensate for cognitive decline. [44] Thus, blue-collar workers, whose jobs place lower intellectual demands on them, are at a disadvantage with regard to cognitive function as compared to white-collar workers. Second, blue-collar work is related to low income, which is associated with poor housing conditions, nutrition, and social environment, potentially giving rise to cognitive impairment [4,36,45]. Third, differences in health-risk behaviors between occupational categories may partly explain the cognitive deficits observed. Individuals who belong to a manual occupational background are more likely to engage in unhealthy lifestyle practices such as smoking, heavy drinking, and physical inactivity [26]. They also have fewer opportunities to engage in psychosocial interaction and receive medical care [46]. In addition to this, blue-collar workers could be exposed to neurotoxic materials at workplace including pesticides, heavy metals, organic solvents, and defoliants [8,37]. Individuals exposed to multi-toxicants are at a higher risk of developing memory impairment and cognitive aging than unexposed workers [8]. 

Our findings indicate that there is a gender-related gap in the degree of association between the longest-held lifetime occupation and cognitive impairment. No significant differences in the risk of cognitive impairment across the occupation were found in men during the 10-year follow-up. This result might be influenced by the fact that men have shorter life expectancy than women [47]. Accordingly, elderly men with poor cognitive function die early and were more likely to dropout. Better and healthier male elderly have high probability of being study participants. Further studies are required to clarify the association between occupation and cognitive impairment. 

On the other hand, female blue-collar workers were at an increased risk of cognitive impairment, and this was sustained even in the ten years of follow-up. In the age group studied, females were disadvantaged in both educational and occupational attainment compared with their male counterparts [48]. Women were given a very low status, and the majority of the oldest women were exposed to a simple environment [49]. Taken together, female blue-collar workers may have been more likely to be exposed to disadvantaged work conditions than male blue-collar workers, and employment in manual work may have played a significant role in the consistent declines in their cognitive abilities [47,49].

This study is focused on cognitive impairment; the cut-point MMSE score of 24 was used. MMSE is the most frequently administered brief cognitive screening method used for identifying dementia [50]. This test is internationally recognized and used not only in research, but also in clinical practice [50]. For persons with MMSE score of 24 or lower, the GPs, with the help of patient’s family members, collected data about the medical history and family history of dementia [51]. However, the use of MMSE as a sole screening modality has been criticized [50]; the score is less sensitive to detect mild impairments (ceiling effect), especially in the highly educated individuals [50]. Furthermore, in those with little formal education and those with severe language problems, the test is insensitive to detect dementia (floor effect) [50]. Other screening tests, such as “Montreal Cognitive Assessment”, “Cambridge Cognitive Examination”, and “Alzheimer’s Disease Assessment Scale-cognitive part” are commonly used in research, clinical, and community setting [52,53,54]. However, there is no agreement about which test should be used as the best approach for early diagnosis of dementia. To estimate strengths and weaknesses, all the above mentioned methods should be comprehensively and systematically assessed in diverse populations and settings [55].

Although previous studies have demonstrated the association between occupation and development of cognitive function impairment, gender-specific analysis has not been conducted despite established gender-based differences in occupational status. Additionally, many previous studies have assessed cognitive function only once [12,21,43]; however, how long the occupational effect lasts remains unclear. The novelty of this study is that blue-collar women were identified to be vulnerable to cognitive impairment in later life, and in these women cognitive impairment persisted for 10 years. Based on our study results, we can suggest several conceptual and practical implications of the analysis. First, our findings indicate the importance of adopting a life-course perspective in designing interventions for preventing cognitive impairment [56]. Accordingly, interventions aiming at mid-life periods might be beneficial especially in the occupational field. Second, although occupational status might not be easily modified, policies reducing exposure to poor residential and work environments (e.g., improve regulations for chemical exposure), building proper social and social cohesion (e.g., investment in community infrastructure), and promoting positive health behaviors (e.g., antismoking programs) may enhance cognitive health of blue-collar workers [57]. Additionally, educational support by the government for improving cognitive health in later life could reduce cognitive disparities [58]. Moreover, if blue-collar workers continue with their education and lifelong learning program elsewhere outside the workplace, it may be expected to promote intellectual stimulation [58].

However, results need cautious interpretation in view of inherent data and methodological limitations and it should be considered. First, they may raise a misclassification bias because the information on the occupation could not fully obtain specific workplace exposure. However, as there are typical workplace exposures for each job type (e.g., non-intellectual demand, low income, and neurotoxic materials at work in blue-collar [4,36,37]; bullying from aggressive customers and emotional fatigue, and mental illness in pink-collar [38]), this study defined the occupational type as approximate workplace exposure. Further studies need to demonstrate the specific occupational exposures that affect cognitive deficits in later life. Second, selection bias may occur when study populations are not representative of target populations. Consequently, the measure of risks would not accurately represent the target population [59]. In this study, selection bias could occur because demographic characteristics were different for the analyzed population and the dropouts. A large proportion of dropouts are composed of attrition during 1st, 4th, and 6th waves and participants without responses in the job history survey. Moreover, subjects with missing values in MMSE scores, income, education, and smoking status were excluded. Thus, the better and healthier elderly have a high probability of study participants and, the prevalence and ORs may be lower than true prevalence and risks in elderly Koreans. In our study, the estimated prevalence of cognitive impairment may be lower than true prevalence, especially in elderly men. The data might be biased because men with an overall better cognitive health condition were most likely included. However, there were no significant differences of follow-ups across the three occupational types for both male (*p* = 0.475) and female (*p* = 0.432) (e.g., the proportion of the dropout across occupation was evenly distributed). Thus, the errors caused by selection bias may be reduced. Third, this study followed the ORs for cognitive impairment for 10 years rather than performing analysis using the comprehensive longitudinal model because there was no information about the exact time-point of cognitive impairment development. This study has a cross-sectional design which cannot be used to test causality. Further studies should clarify the longitudinal changes of cognitive function. Fourth, our study used simplified job classification which cannot reflect specific occupational aspects. Although detailed classifications (e.g., physical demand or complexity of job) have been used in this field, available job information cannot detect all sophisticated occupational characteristics. Fifth, to obtain data related to the longest-held lifetime occupation, participants in the KLoSA were asked to think back over their lives, potentially giving rise to recall bias. However, the method of data collection relied upon the filling calendar to help boost participants’ memory. Thus, the probability of misclassification bias associated with lifetime occupation was minimized. Sixth, the results of our study cannot be generalized to the entire Korean population aged over 65 because the survey excluded institutionalized elderly. In our data, 20% of female participants were housewives who had never been employed, and were accordingly excluded from our analysis. Therefore, further research on cognitive decline in the unemployed population is required. Seventh, the duration of the longest-held lifetime occupation in males was approximately 10 years longer than in females (40.05 years versus 31.78 years). Thus, attempts to compare the impact of the longest-held lifetime occupation on cognitive impairment between the genders should be undertaken with caution. Eighth, although the KLoSA surveys are biennial and data for all relevant years in the period from 2006 to 2016 were available, we only included data from 2006, 2012, and 2016. Had we used all the data from 2006 to 2016, more than half of the baseline sample would have been ruled out; including only those followed up in 2006, 2012, and 2016 led to the lowest possible attrition rate (51.68%). Ninth, there may be other unmeasured confounders, such as dietary intake, digestive health problems, and sleep quality and duration, which could not be adjusted for in our analysis. Further studies are needed to consider these confounding factors for clarifying the association in the analysis. Finally, although the K-MMSE is an easy measure of cognitive functioning and is widely used for screening early dementia, it is insensitive to detecting early dementia because it causes false negative rates and has negative predictive power [60,61]. Thus, further studies are needed using a more accurate tool for assessing cognitive function in the elderly. 

## 5. Conclusions

In conclusion, the longest-held lifetime occupation was a significant discriminator with regard to cognitive impairment in later life. No significant association between cognitive impairment and the longest-held occupation in a lifetime was observed in males. In female older adults, however, the disparities in cognitive impairment remained significant even after 10 years. This study suggests that the longest-held lifetime occupation should be taken into consideration when screening cognitive decline in the elderly, especially females. 

## Figures and Tables

**Table 1:** Characteristics of the study population according to the longest-held lifetime occupation by gender.

	Male				Female			
	Blue-Collar	Pink-Collar	White-Collar	*p*-Value	Blue-Collar	Pink-Collar	White-Collar	*p*-Value
	*n* = 473 (64.27%)	*n* = 75 (10.19%)	*n* = 188 (25.54%)		*n* = 743 (74.52%)	*n* = 200 (20.06%)	*n* = 54 (5.42%)	
Age								
Mean (SD)	70.23 (4.33)	69.41 (4.56)	70.92 (4.51)	0.0325	71.16 (4.98)	70.64 (5.09)	69.04 (3.37)	0.0059
Marital Status								
Married	442 (93.45)	66 (88)	178 (94.68)	0.2898	376 (50.61)	98 (49)	34 (62.96)	0.3743
Divorced/Widowed	30 (6.34)	9 (12)	10 (5.32)		364 (48.99)	102 (51)	20 (37.04)	
Never Married	1 (0.21)	0 (0)	0 (0)		3 (0.4)	0 (0)	0 (0)	
Education								
Elementary or Less	306 (64.69)	28 (37.33)	23 (12.23)	<0.0001	703 (94.62)	166 (83)	12 (22.22)	<0.0001
Middle/High School	160 (33.83)	42 (56)	95 (50.53)		40 (5.38)	32 (16)	36 (66.67)	
College or Higher	7 (1.48)	5 (6.67)	70 (37.23)		0 (0)	2 (1)	6 (11.11)	
Income (Yearly, 10,000 KRW) *								
<1000	304 (64.27)	43 (57.33)	103 (54.79)	0.051	519 (69.85)	138 (69)	34 (62.96)	0.7943
1000–2500	117 (24.74)	26 (34.67)	54 (28.72)		159 (21.4)	41 (20.5)	14 (25.93)	
>2500	52 (10.99)	6 (8)	31 (16.49)		65 (8.75)	21 (10.5)	6 (11.11)	
Cigarette Smoking								
Never Smoker	206 (43.55)	33 (44)	87 (46.28)	0.0003	716 (96.37)	190 (95)	53 (98.15)	0.8463
Former Smoker	108 (22.83)	19 (25.33)	68 (36.17)		6 (0.81)	2 (1)	0 (0)	
Current Smoker	159 (33.62)	23 (30.67)	33 (17.55)		21 (2.83)	8 (4)	1 (1.85)	
Alcohol Drinking								
Current Drinker	275 (58.14)	42 (56)	105 (55.85)	0.8625	91 (12.25)	27 (13.5)	5 (9.26)	0.0371
Former Drinker	70 (14.8)	9 (12)	30 (15.96)		20 (2.69)	0 (0)	3 (5.56)	
Nondrinker	128 (27.06)	24 (32)	53 (28.19)		632 (85.06)	173 (86.5)	46 (85.19)	
Physical Activity								
Yes	152 (32.14)	33 (44)	138 (73.4)	<0.0001	158 (21.27)	74 (37)	29 (53.7)	<0.0001
No	321 (67.86)	42 (56)	50 (26.6)		585 (78.73)	126 (63)	25 (46.3)	
Muscle Strength **								
Low	226 (47.78)	29 (38.67)	68 (36.17)	0.0159	363 (48.86)	80 (40)	18 (33.33)	0.0123
High	247 (52.22)	46 (61.33)	120 (63.83)		380 (51.14)	120 (60)	36 (66.67)	
Chronic Disease								
Yes	105 (22.2)	13 (17.33)	40 (21.28)	0.633	169 (22.75)	54 (27)	9 (16.67)	0.2241
No	368 (77.8)	62 (82.67)	148 (78.72)		574 (77.25)	146 (73)	45 (83.33)	
Living Alone								
Yes	311 (65.75)	50 (66.67)	118 (62.77)	0.7336	453 (60.97)	105 (52.5)	38 (70.37)	0.0252
No	162 (34.25)	25 (33.33)	70 (37.23)		290 (39.03)	95 (47.5)	16 (29.63)	
Well-Being								
Yes	382 (80.76)	63 (84)	174 (92.55)	0.0009	564 (75.91)	152 (76)	45 (83.33)	0.4605
No	91 (19.24)	12 (16)	14 (7.45)		179 (24.09)	48 (24)	9 (16.67)	
Work Duration †								
Low	168 (35.52)	66 (88)	146 (77.66)	<0.0001	305 (41.05)	160 (80)	40 (74.07)	<0.0001
High	305 (64.48)	9 (12)	42 (22.34)		438 (58.95)	40 (20)	14 (25.93)	
Time Spent after Retirement ‡								
Low	286 (60.47)	47 (62.67)	48 (25.53)	<0.0001	399 (53.7)	93 (46.5)	24 (44.44)	0.1057
High	187 (39.53)	28 (37.33)	140 (74.47)		344 (46.3)	107 (53.5)	30 (55.56)	
K-MMSE Score								
Mean (SD)	24.90	26.43	26.53	<0.0001	21.10	23.15	26.13	<0.0001

* <10,000,000 KRW (approximately <9000 USD), 10,000,000e 25,000,000 KRW (approximately 9000–22,500 USD), or >25,000,000 KRW (approximately 22,500 USD). ** Muscle strength was categorized based on gender-specific handgrip strength median values; high (male >28.75 kg and female >17.0 kg) or low (male ≤28.75 kg and female ≤17.0 kg). † Work duration was categorized based on gender-specific median values; high (male >39 years and female >29 years) or low (male ≤39 years and female ≤29 years). ‡ Time spent from retirement to examination of MMSE was categorized based on gender-specific median values; high (male >1 year and female >8 years) or low (male ≤1 year and female ≤8 years).

**Table 2:** The odds ratios (ORs) of cognitive impairment (MMSE < 24) in male elderly over 10 years (2006, 2012, and 2016).

	2006		2012		2016	
**Unadjusted Model**	**OR**	**95% CI**	**OR**	**95% CI**	**OR**	**95% CI**
White-Collar Job	Reference		Reference		Reference	
Blue-Collar Job	3.34	(2.04–5.48)	2.02	(1.36–3.00)	2.16	(1.49–3.12)
Pink-Collar Job	0.95	(0.40–2.25)	1.1	(0.58–2.09)	1.46	(0.82–2.60)
**Adjusted Model †**	**OR**	**95% CI**	**OR**	**95% CI**	**OR**	**95% CI**
White-Collar Job	Reference		Reference		Reference	
Blue-Collar Job	1.69	(0.88–3.24)	1.13	(0.65–1.95)	1.34	(0.80–2.26)
Pink-Collar Job	0.68	(0.26–1.80)	0.94	(0.44–2.00)	1.24	(0.62–2.48)

† The model was adjusted for age, marital status, education, income, cigarette smoking, alcohol drinking, physical activity, muscle strength, chronic diseases, job duration, time passed from retirement to assessing cognitive function, well-being, and living alone.

**Table 3:** The ORs of cognitive impairment (MMSE < 24) in female elderly over 10 years (2006, 2012, and 2016).

	2006		2012		2016	
**Unadjusted Model**	**OR**	**95% CI**	**OR**	**95% CI**	**OR**	**95% CI**
White-Collar Job	Reference		Reference		Reference	
Blue-Collar Job	8.13	(3.91–16.88)	3.56	(1.99–6.38)	4.82	(2.66–8.73)
Pink-Collar Job	4.35	(2.02–9.36)	1.86	(1.00–3.48)	3.11	(1.64–5.89)
**Adjusted model †**	**OR**	**95% CI**	**OR**	**95% CI**	**OR**	**95% CI**
White-Collar Job	Reference		Reference		Reference	
Blue-Collar Job	2.49	(1.05–5.88)	1.57	(0.74–3.31)	2.17	(1.02–4.65)
Pink-Collar Job	1.96	(0.81–4.78)	0.93	(0.43–2.02)	1.89	(0.86–4.16)

† The model was adjusted for age, marital status, education, income, cigarette smoking, alcohol drinking, physical activity, muscle strength, chronic diseases, job duration, time passed from retirement to assessing cognitive function, well-being, and living alone.
